# Qualitative study of acceptability, benefits, and feasibility of a food-based intervention among participants and stakeholders of the RATIONS trial

**DOI:** 10.1371/journal.pgph.0004219

**Published:** 2025-04-28

**Authors:** Sunita Sheel Bandewar, Madhavi Bhargava, Hema Pisal, Sharanya Sreekumar, Anant Bhan, Ajay Meher, Anurag Bhargava

**Affiliations:** 1 Secretary General, Forum for Medical Ethics Society, Mumbai, India; 2 Professor, Department of Community Medicine, Yenepoya Medical College, Mangalore, India; 3 Center for Nutrition Studies, Yenepoya (Deemed to be University), Mangalore, India; 4 Independent Researcher and Anthropologist, Pune, Maharashtra, India; 5 Program Assistant, Forum for Medical Ethics Society, India; 6 Global Health and Ethics Researcher and Consultant, Forum for Medical Ethics Society, Mumbai, India; 7 Senior Programme Manager and Team Lead, Swasthya Swaraj Society, Kalahandi, Odisha; 8 Professor, Department of Internal Medicine, Yenepoya Medical College, Mangalore, India; 9 Department of Medicine, McGill University, Montreal, Canada; Emory University Rollins School of Public Health, UNITED STATES OF AMERICA

## Abstract

A qualitative study was conducted during the RATIONS trial to explore the perceptions, experiences, and expectations of participants and stakeholders on the acceptability, benefits, and feasibility of the nutritional intervention to complement the trial findings for deeper exploration into why and how of these findings and other allied themes. Using purposive sampling, we recruited 58 individuals for 22 in-depth interviews (IDI) and four focus group discussions (FGDs) between January and June 2022. These included 12 patients with TB, six household contacts, and other stakeholders (18 trial members, 18 government community workers, and four National TB Elimination Program (NTEP) staff). All IDIs and FGDs were audio-recorded, transcribed, and translated. The codes were generated using an inductive process and categorized manually into themes, with direct quotes describing the themes. The intervention was found to be acceptable in terms of cultural compatibility, quality, quantity, and duration; considered beneficial in helping tolerate the adverse effects of medications, weight gain, and resuming work; and was considered life-saving by many during the COVID-19 pandemic. Other observations included food-sharing in the control arm, inability to regain pre-disease functional status despite weight gain, and preference for in-kind support. Community health workers expressed confidence in its feasibility and willingness to take responsibility for its implementation. The NTEP staff considered it feasible if necessary resources were provided. This qualitative inquiry reflected the perspectives and lived experiences of households experiencing poverty, food insecurity, TB and the stakeholders serving them. Their voices are relevant in framing policy and practice in the NTEP and future research in India and similar low-resource settings. The food-based intervention was perceived as acceptable, feasible, and beneficial for the recipients and the NTEP. Opinion on cash or support in kind was divided; many preferred in-kind support over cash, but others expressed a requirement for both.

## Introduction

Tuberculosis (TB) is one of the most widely prevalent infections, affecting 23% of the global population [1]. An estimated 10.8 million people fell ill with TB in 2023, with 1.25 million dying from TB in 2023, the highest with any single pathogen [2]. As a quintessential social disease, poverty is a major factor that affects both the exposure to M. tuberculosis and the outcomes of TB infection [3]. Undernutrition is an important factor associated with poverty that mediates its risk of active TB, leading to immunological dysfunction, referred to as nutritionally acquired immunodeficiency syndrome [4]. It is the leading risk factor for TB incidence worldwide, accounting for as many cases annually as due to HIV and diabetes combined [2]. Also, undernutrition is a widely prevalent comorbidity in persons with TB (PwTB) and is a consistent risk factor for TB mortality [5]. A Cochrane review was inconclusive about the effect of nutritional supplementation on treatment outcomes in PwTB [6]. However, the WHO recommended nutritional assessment and counseling for all PwTB and support to some groups as integral components of TB care [7].

The Reducing Activation of Tuberculosis by Improvement of Nutritional Status (RATIONS) trial, a cluster-randomized trial of nutritional intervention in PwTB and their HHCs, in a setting with a high prevalence of poverty, food insecurity, undernutrition, and a low prevalence of HIV and drug-resistant TB, addressed this evidence gap [8]. This was the first trial globally to estimate the effect of nutritional supplementation on TB incidence in a group at high risk of developing TB [9]. The trial population consisted of 2800 PwTB and 10,345 HHCs [9,10]. For ethical reasons, the documented high prevalence of severe undernutrition in PwTB in India and recommendations for nutritional care in a national policy document [11–13], PwTB in both trial arms received a monthly food basket and micronutrients for the treatment period (Table 1). The HHCs in the control arm (1400 families) received a food basket and micronutrients based on family size for the treatment period of the index PwTB. The primary outcome was the difference in the TB incidence in the HHCs in both arms over a two-year follow-up period [9]. Secondary outcomes included the nutritional and clinical outcomes [10]. This trial was registered with CTRI-India (CTRI/2019/08/020490).

**Table 1:** RATIONS Study intervention.

	Intervention arm	Control arm
Index case (1200 Kcal of energy and 52 gm proteins)	5 kg of rice 3 kg roasted Bengal gram powder (locally called as *sattu*) 1.5 kg of milk powder 500 ml vegetable oil One RDA of micronutrient	5 kg of rice 3 kg roasted Bengal gram powder (locally called as *sattu*) 1.5 kg of milk powder 500 ml vegetable oil one RDA of micronutrient
Household contact	5 kg rice 1.5 kg pulses (split pigeon peas) One RDA of micronutrient per adult/adolescent HHC Half of this for children less than 10 years.	Nutritional counseling Usual food assistance through the public distribution system

Source: RATIONS Protocol [8]; RDA = Recommended Dietary Allowance.

This paper reports the findings from a qualitative sub-study of the RATIONS trial. The sub-study assesses trial participants’ and stakeholders’ perceptions, experiences, and expectations on the acceptability and feasibility of this food basket-based intervention. These have relevance to guideline development for programmatic implementation and future research [14].

## Methodology

### Study design

### Study setting and context

The trial was conducted in association with the National Tuberculosis Elimination Program (NTEP) in four districts of Jharkhand in eastern India [8]. Jharkhand (“land of forest”) is rich in mineral resources and has a significant forest cover and a hilly terrain. More than three-quarters of its population lives in rural areas, and more than a quarter comprises scheduled tribes (ST), a marginalized set of communities. Two-thirds of the participants in the trial were tribals, reflecting their higher proportion in the selected districts.

Jharkhand has many tribal groups: the Mundas, Gonds, Santhals, Ho, Oraons, and Bhumij [15]. They continue to fare the worst in terms of income, education, health, nutrition, prevalence of TB, access to health services, and nearly half are classified as suffering from Multidimensional poverty [16]. Infant and under-five mortality, nutrition indicators like wasting and stunting in children, and thinness and anemia in adults are higher compared to other social groups [17]. The World Bank reported that 55% of tribal households had some degree of food insecurity throughout the year [18]. The prevalence of TB in tribals is more than double the national average (703/100,000 vs. 316/100,000), and in some communities like the Sahariyas, it is almost 10 times [19–21]. In Jharkhand, agriculture is the backbone of the rural economy, revolving around a single rain-fed rice crop. Food insecurity is worse in the pre-harvest months of September to November due to small land holdings and subsistence farming [18].

### Participant types

The participants in the qualitative sub-study (Table 2 and S1 Table) included the PwTB and their HHCs equally represented from the intervention and the control arms, the Sahiyas, the NTEP staff, the trial field staff that anchored the regular follow-up of the families, and the food delivery, two project consultants who supervised the field staff through field visits, checked food stocks, prepared the monthly rations procurement order, and liaised with government program staff. Sahiyas ("female friend" in the local language) is the local name for Accredited Social Health Activists (ASHAs), the community health workers associated with the National Health Mission [22]. Among many preventive and promotive health activities they conduct, the TB component includes case-finding, facilitating diagnosis and treatment, and enrolment in the scheme for direct benefit transfers (DBT), a monthly cash transfer for all PwTB under the Nikshay Poshan Yojana (NPY) to support nutritious food during treatment [23].

**Table 2:** Participant profile of the qualitative sub-study of the RATIONS trial.

Participant characteristics (N = 58)		N (Percent)
Age group	18-29	13 (22.4)
	30-39	32 (55.2)
	40-49	4 (6.9)
	50-60	9 (15.5)
Sex	Male	34 (58.6)
	Female	24 (41.4)
Education	Not adequately literate	6 (10.4)
	Primary School	5 (8.6)
	High School	21 (36.2)
	Graduate	16 (27.6)
	Post Graduate	8 (13.8)
	Not mentioned	2 (3.4)
Occupational status	Unemployed	8 (13.8)
	Employed	50 (86.2)
Type of participants	Patients with TB	12 (24.1)
	Household contacts*	6 (10.3)
	RATIONS field staff	16^#^ (25.9)
	Sahiyas	18 (31.06)
	RATIONS Project consultants	2 (3.4)
	NTEP Staff	4 (1.8)
Type of data collection (n = 26)	In-depth interview	19 (73.1)
	Focused group discussion^@^	4 (15.4)
	Group interview	3 (11.5)

* 2 Household Contacts were also Sahiyas; ^#^1 field staff was also a TB survivor; ^@^ 6–10 Sahiyas and 8–10 field staff present in each FGD.

### Research team attributes

The qualitative sub-study was anchored by social scientists from the Forum for Medical Ethics Society (FMES) with extensive experience in qualitative research and ethics, uniquely providing a third-party independent perspective. The lead trial investigators had experience spread over two decades of working with marginalized tribal communities in central India and researching interactions between TB and nutrition [24]. Their long-term engagement with the NTEP, involvement in the trial conceptualization and its conduct, and experiences with the implementation of the intervention helped the stakeholder mapping, framing the in-depth interview (IDI) and focus group discussion (FGD) guide, and interpreting the data.

### Sample size

The eligibility criteria for this study included those above 18 years of age, trial participants (PwTB or their HHC), or stakeholders in the RATIONS/NTEP who agreed to participate in this qualitative study. The sample included an equal number of participants from each arm of the trial, with the representation of households from urban, rural, and remote locations as well as different communities. The trial participants were relatively homogeneous, with more than two-thirds belonging to indigenous communities with similar problems of poverty and food insecurity and challenged by a similar disease and its consequences. We ceased to recruit participants when it was felt that no additional themes or insights were emerging and data saturation was achieved. The qualitative sub-study was located within a large cluster randomized nutritional intervention trial with a substantive quantitative component. The qualitative sub-study was conducted within the context of quantitative information emerging from the trial.

### Sampling and recruitment strategy

The study used a purposive sampling framework with participants and stakeholders across urban/rural geography, community groups, and social class. The trial field staff helped recruit PwTB and their HHCs and Sahiyas, while the trial investigators helped recruit the field staff and project consultants. The NTEP program staff were recruited with inputs from the trial team. None of the participants declined participation.

### Data collection techniques and tools

The RATIONS trial ended in August 2022, and the data collection for qualitative inquiry was done from January to June 2022. The overall contours of the semi-structured interview guide were adapted suitably for the respective stakeholders. The interview guide was designed in English; however, the interviews were conducted in Hindi. The IDIs of trial participants occurred at their residences, and all FGDs were in community halls (the Panchayat Bhawan) or district headquarters. Participant IDIs began with general questions followed by probes when required (S1 Text). The general questions included, “Can you tell me about your experience as a PwTB/HHC/RATIONS project consultant/NTEP staff? Can you tell me about the food basket? and can you tell me about your main challenges?” The FGDs began with general questions followed by probes when required (S2 Text). These included, “Can you tell me your role in TB care? What do you know about the RATIONS project? How are you engaged in it? and can you tell me about the challenges of RATIONS-like food-based intervention?”

During data collection in the field, all the prevailing infection prevention guidelines of the COVID-19 pandemic were followed. All data collected was face-to-face except online IDIs with the two project consultants and one with senior NTEP staff. All interviews were conducted in Hindi, and in some, the aid of a local interpreter was utilized, and leading questions were avoided as far as possible. The duration was 90 to 120 minutes for IDIs and up to 180 minutes for FGDs.

### Data processing and analysis

Interviews were continued till HP and SS identified data saturation. All audio-recorded interviews were transcribed verbatim, translated into English by SS, and cross-checked by SSB. Both repeatedly read these to identify any grammatical errors or consistency of the meaning. Additionally, investigator triangulation was used to understand some of the technical terms related to TB and NTEP and to improve the credibility and validity of the results. This was done by consistently discussing and reconciling the context of keywords, broader concepts, and terminologies among the research team throughout the data collection process. For the analysis, similar responses were coded and grouped. Codes were generated using an inductive process and manually categorized by the social scientists in the team into themes and sub-themes. Direct quotes were employed to describe the themes.

### Ethics

Written informed consent was obtained from all participants: the PwTB, HHCs, project staff, the Sahiyas, and the NTEP staff. Appropriate privacy and confidentiality measures were undertaken, and data were anonymized. The RATIONS trial received ethics approval from the Indian Council of Medical Research – National Institute of Research in Tuberculosis ethics committee (ICMR-NIRT: 2018020), Chennai. Only FGD participants were provided refreshments due to its time-intensive nature, and there was no monetary compensation for participation.

### Measures and techniques to enhance the quality of data

Any RATIONS trial team member known to the trial participants was not part of the IDI to ensure rigor and credibility. Similarly, during the FGDs with Sahiyas, no government health staff were present. The lead investigators recused themselves during FGDs and IDIs of the field staff and project consultants.

## Results

The stakeholders’ perspectives that emerged consisted of not only those related to the stated objectives like ascertainment of acceptability, content of food basket, and its benefits in terms of weight gain and ability to work but also the unstated aspects of the intervention. These included positive experiences of being cared for, well-being, personhood, and dignity. The results are organized around the themes that emerged in the content analysis and supported by relevant quotes, also represented in Table 3.

**Table 3:** Themes of the qualitative inquiry in RATIONS study and representative quotes.

Theme	Representative quote	Participant
Acceptability of the food basket	“We are Munda. We have Munda, Gopa, Kurmi, Lohar (artisans engaged with fabrication and ironwork), and Santhals in this village. Eating habits (of all these ethnic/ artisan groups) are almost the same but (with) some difference.”	Male PwTB
Perceived benefits of the food basket	“My mother became better soon with ration. We did not find it difficult to give her the food she needed. She was very weak, but after getting food, her weight increased.”	Female HHC
Quantity and content of food basket	“It is good, but some patients say it would have been better if eggs had been given.”	Sahiya
Duration of support	“It is given for the patient’s welfare, so it can be stopped when the patient becomes healthy. But if the patient is not okay, it should be prolonged.”	Female PwTB
Resource constraints in TB-affected households	“…..we would not pay attention to our diet as we go to work and sometimes sleep without food only.”	Male PwTB
Food insecurity and the COVID-19 pandemic	“COVID-19-related lockdown worsened the livelihood and food availability. Our patients badly needed the food basket, but the police used to drive the field staff away initially (during lockdown). It was tough.”	Male project consultant
Feasibility, mainstreaming, equity, including cash vs. kind	“Send the food ration according to the number of patients in each village and drop it off at the *Panchayat office.* We will collect it and deliver it to the patients. At village level it is easy.” “If I were to choose, I would choose food over cash. If you want to give both, that is also fine”	Sahiya
Stigma	“All this (stigma) is present among urban people, not in our villages”	Male Medical Officer, NTEP

### 1 . Acceptability of the food basket

We explored the participants’ views regarding the acceptability of the food basket, palatability, ease of cooking, and quality compared to their usual food, as described below.

#### 1.1 Cultural compatibility.

Almost all participants asserted cultural compatibility of the intervention, along with good taste. Some mentioned that ‘sattu,” although available in the market, is unaffordable and was mainly for special occasions/festivals.

*“I liked it all. … I used to make sattu balls/laddu and eat. … Milk powder, I mixed water; I made rice and used oil to cook vegetable curry.”* (Female PwTB)

The diverse ethnic communities have their own specific food-related cultural practices. They have certain similarities and differences regarding their food preferences. Several meat items were desirable inclusions in the interactions with varied preferences across ethnic communities. This and certain commonalities justified the content of a “desirable” food basket.

*“Like some (ethnic groups) eat suvar (pork) and some don’t. But most eat meat, fish, and eggs … (Therefore,) the food basket could be the same for all. All consume eggs. Santhal, Munda, Mahato, Karmakar, and even Brahmins eat eggs. (And) meat, fish can be there, but not all eat it.” (Male PwTB)*

#### 1.2 Quality of the food basket, its comparison with their regular food.

All trial participants unambiguously expressed satisfaction with the quality of the food basket. Many felt it was far better than what they usually eat and mentioned that the public distribution system (PDS) must also provide additional grocery items.

*“What they gave was good and tasty. We get sattu in the market, but the sattu they gave we won’t get in the nearby shop, not as good.”* (*Male PwTB*)

We further probed the quality by comparing food baskets with PDS food, one of the largest social welfare schemes of fair-priced government shops on which many poorer families depend across India. These insights are relevant as PDS can be a good strategy for the scale-up of the intervention in the future. Broadly speaking, three distinctive factors emerged in this comparison: the diversity of food items, the quantity, and the quality.

“*There is much difference between the rations we receive from the PDS and what they gave. (For example), they (PDS) only give rice, and here (in the trial food basket) we get sattu, milk and better rice*.” (*Male PwTB*)

### 2 . Perceived benefits of the food basket

#### 2.1 Improvement in weight and strength.

The expression of benefits and gratitude varied according to the participant’s needs, vulnerabilities, and lived experiences. An important message underscored a positive experience of feeling better, gaining weight, and gaining strength.

*“I was a very thin person earlier. This food was good for me as it increased, almost doubled my weight.”* (*Female PwTB, physical disability, Indigenous community).*

The benefits went beyond ‘feeling better’ to positively impact other spheres of life, such as schooling, sports, and a sense of well-being.

*“I had stopped my studies and playing on the ground when I had the disease. Now, I have started to play volleyball*.” (*Male PwTB, youth*)

#### 2.2 Better adherence to treatment due to lesser side effects.

Participants drew a link between “powerful” TB medicines and the favorable impact of adequate good food from their lived experiences. Many highlighted that TB medicines could have been difficult to consume without the food basket, essential for treatment adherence, vital to achieving a cure, and important programmatic implications.

*“If I had not got the ration, it would have been tough for me. The medicines are so powerful that I would have died.*” (*Male PwTB*)

The complex relationship between undernutrition, TB, side effects of medicines, lack of compliance, and drug resistance was explained by a senior medical officer of tribal origin who has been working in the region for > 25 years:

*“Side effects of TB medicines are more in malnourished patients. After some time, he will stop eating food due to vomiting. We will know about it only when we do the follow-up, and then we will restart medicines. This irregularity would ultimately develop drug-resistant TB.” (Male Medical Officer)*

#### 2.3 Effects on outcomes like death and recurrence of TB.

The food basket played a role in patient-centered outcomes. Moreover, these are also important from the NTEP perspective: TB deaths and recurrence. The Sahiyas, who were uniquely placed to compare patients within and outside the trial, shared their observations regarding the benefits of the food basket.

*“Those who received food baskets improved completely. They gained weight. Another patient on treatment migrated somewhere, but he died there. … All those who received food baskets are doing well now, but those who did not receive them have got TB again.” (FGD Sahiyas)*

#### 2.4 Other collateral benefits: the positive community impact.

We noted some collateral benefits of the trial team’s engagement with the families. For example, a HHC developed TB and was not eligible for a food basket. However, due to the knowledge gained about the type of diet that would be beneficial, the family attempted to maintain a diet like the trial diet. The positive impact on community food practices, thus, goes beyond TB. The home visits by the field staff and periodic checkups were added benefits.

“*We would not have come to know about all this, and we would not have this knowledge, such as how TB occurs, how it spreads, what food is needed to prevent it, and which medicine we should take for this disease.”* (*Male HHC)*

The lived experiences of individuals brought unique insights into how they perceive the relevance of such an intervention.

*“When I had TB, we had to go to STS (Senior Treatment Supervisor), and he would reply to our questions. But no one visited our house to help us understand diet and lifestyle, not when I had TB.”(female field staff and TB survivor)*

### 3. Expectations regarding the food basket: quantity and content

During the interaction with patients, their families, and Sahiyas, various expectations regarding the content of the food basket were expressed for a possible roll-out on a large scale. Eggs and non-vegetarian items were often included in this wish list.

*“I will take potato, all kinds of vegetables such as tomato, brinjal then dal and rice. If possible, I would like to have meat (once) in a week.*” (*Male PwTB*)

Some other Sahiyas mentioned during the same FGD “potatoes, sugar-flavored branded supplements, soyabean.” But then immediately, someone in the group quipped that if we demand more, even those getting the food basket will not get it. About the quantity, most felt it was okay, and some wanted more. The trial participants in the control arm appeared to share with others, especially with children.

*“The quantity of ration should vary by region. People in the village work hard and need more food. In cities, they sit in the workplace and drink milk and nutritious food, but village people do manual work and eat less nutritious food.” (Female HHC)*

### 4. Duration of support, regaining strength, but not enough to resume work

Several study participants expressed that although the food basket was helpful and they had recovered, they have not been able to resume work, often labor-intensive, primarily because they continue to feel weak.

*“I was a mason. I felt as if having gained strength but not as much as before disease. The ration provided surely helped improve my health. … but I feel weak even now and am just staying at home.*” (*Male PwTB*)

The food basket was responsive to their nutritional needs, and the benefits transcended TB recovery to improve health, but at times, not sufficient to resume their earlier work. Considering the high prevalence of severe undernutrition in PwTB in the trial, a significant proportion remained underweight at the end of treatment. The food basket was extended for these patients.^10^ A patient with TB and diabetes who was underweight and regained normal weight put it across very well,

“*if a person becomes healthy, then they can stop giving ration, but if someone is still weak, then they should continue to give them the food for their health.”(Male PwTB)*

A reflection of the understanding of the complexity of such an intervention by the government, the extent to which it can be done, and the general sense of resource constraints of public spending were evident in the expression that they do not wish to receive the support for a protracted period than the health of the patient warrants.

### 5. Resource-constrained circumstances of households affected by TB

We encountered trial participants who did not consume milk regularly. Often, participants had limited food items or only one or two meals during the day and nothing else.

*“We don’t have not much. I ate only one or two meals and used to eat very little then. (Before receiving the food basket) My meal used to be Alu ka sabji (potato side dish), rice (only), and this much (showing with hand).” (Male PwTB)*

Several trial participants rarely included sattu and milk in their diet. Some mentioned reasons like limited purchasing capacity, but many did not mention any specific reasons, with an unstated dignified silence that did not escape the interviewers. Limited financial resources, dependence on contractual work, often involving heavy manual labor, and overall subsistence-based livelihoods implied precarious living conditions that can quickly worsen with TB. Loss of livelihood and poor strength to resume work with no reserve to sustain the difficult times worsened the situation for these families. This became especially evident when an incident TB case developed in a household and the intervention could not be provided to them, and the DBT was delayed.

*“In my house, there was very little food. Our didi (Sahiya) and everyone else said I must eat good food because medicine is powerful. They said I would get money (not ration). That also I did not get till now.”* (*Male HHC)*

### 6. Food insecurity and the COVID-19 pandemic

With the loss of livelihood and lockdown, the pandemic worsened the already precarious conditions for the community. It shaped the views on the intervention for the trial participants and the field staff who faced many difficulties during the pandemic.

*“We had to give food to my father on time, and we had also to feed everyone in the family. So, this food was lifesaving.” (Female HHC)*

*“I like to say that during COVID, people used to say that they survived because of this food; otherwise, we would starve. In the RATIONS trial, we could see sensitive issues such as hunger and poverty very closely.”(Male field staff).*

### 7 . Feasibility, mainstreaming, and equity

We spoke with the Sahiyas, field staff, and NTEP staff about possible feasibility issues, and many were willing to contribute as they felt it was essential despite the challenges.

#### 7.1 Sahiyas.

The Sahiyas were willing to get involved with the logistics if this was rolled out at scale. However, they mentioned that there should be good supportive supervision and monitoring as they do not want to be accused of pilferage.

**“** *As Anganwadi workers (India has an Integrated Child Development Services Scheme where preschool children have informal education and supplementary nutrition, among other things) bring their goods, we will also unite to do the same. We are 4 in a village and will come together and do it in a tempo (small goods carrier vehicle). We will do anything for the patients.”(Sahiyas)*

#### 7.2 Field staff of the trial.

Apart from food delivery, the connection of the field staff with their assigned 80–120 households was an important aspect of care. This takes the issue from beyond feasibility to humane care for patients with problems like TB, where patients often experience stigma and discrimination.

“*One patient, when I took his arm to take BP, he started crying. When I asked him about it, he said that the doctor never touched him in the hospital. He made me sit far away and talk and prescribed medicines to me from a distance. You are the first person who has touched and is talking to me face to face.”(Male field staff)*

The field staff expressed difficulty distributing the food basket on top of the rigorous data collection for a research trial. During the peak of the trial, with enrolment and follow-up of already enrolled patients, the difficult-to-reach areas or the scattered hamlets were especially challenging. But gratification was also expressed by most.

*“The geographical situation here is such that the villages are scattered. So how many people can you visit in one day? But it feels good when you do something for someone….. Every job provides you money, but only a few provide ultimate pride and satisfaction.” (Female field staff)*

For feasibility, it is essential to mention the acceptability of project field staff delivering food baskets at the doorstep. While most participants were happy to have it delivered, there were challenges initially with some families. Villagers would gather and ask many questions due to trust issues. Things improved gradually with the improvement in the health of the trial participants, appropriate explanations by Sahiyas, and other sociocultural methods employed by the field staff.

“*We drank their water, ate food that they offered, and they start trusting us once we help them in an illness and facilitate investigations.”(Male field staff)*

#### 7.3 NTEP staff.

The feasibility aspect was also discussed with the NTEP staff, if the intervention was scaled up to include all PwTB at the district level. While most agreed that this is needed, a senior NTEP staff foresaw challenges of procurement, storage, and distribution, which can be overcome by budgeting appropriately for human resources

“*Even if done on a big scale, I do not think it should cause many problems because patient numbers will slowly reduce (with this intervention).”(Male STS)*

#### 7.4 Who should receive? The equity lens.

The equity aspect of who should get it was discussed in various ways: whether food should be given to only microbiologically confirmed PwTB, all PwTB, or their entire households. The responses brought to the fore the challenges that the NTEP staff can face if it is done selectively, especially in food-scarce settings and the foreseeable issue of food-sharing. The Sahiyas expressed that it was uncomfortable for them that some patients who were part of the trial received food and others did not:

*“All patients should get food. If food is given to all, the patient will recover quickly, and the disease will not spread. Most of them are poor, so all should get.”(Sahiya)*

Food sharing was especially common in the control arm, with a female index patient, when there were toddlers or more children in the family. One of the grandmothers, who was also a patient, said,

“*I give the milk powder to my grandson when I have it” (Female PwTB*)

For extending food to HHCs, the responses were “yes” and “no,” each qualified appropriately*.*

*“No, the nutritious food is for the patient’s good health; they are taking medicines, and there are side effects. Why give it to the whole family?” (Female HHC).*

On further probing these divergent opinions, the choices and preferences were rooted in the understanding of economic constraints. The resource constraints and possible non-feasibility for the government in supporting the food provision for the entire household of all PwTB might explain this divergence in the response. This was despite their own microcosms against the backdrop of difficult personal circumstances and limited financial capabilities.

#### 7.5 Cash and/or kind.

Most participants felt that both cash and food in kind are important. But when asked to choose, many felt that food is better than cash, which may get spent on things other than nutrition. The divergent opinion was also based on the presumption that patients will use the money on unnecessary expenses, including the possibility of alcohol. But at the same time, cash could be used for the food of their choice.

*“Considering the present situation, Rs 500/- is not much for the patients. Maybe this amount needs to be increased.” (Male STS)*

*“I would choose food and medicine. To get well, we need medicine, and to become healthy, we need food. It will be difficult for TB patients to go outside and buy the food items needed for their health.” (Male PwTB)*

However, it is important to note that branded and chocolate-flavored powders advertised as energy boosters were commonly mentioned during many interviews.

### 8. Stigma

Stigma can be reflected in keeping distance from TB patients and their families, and at times, it could even lead to ostracism – subtle or obvious. Stigma was more prominent in urban areas than in rural and tribal communities. Insights from one of the field staff, also a TB survivor, were helpful.

*“A girl with TB was of marriageable age, and the family did not disclose this as it would hamper her prospects of marriage. We were told not to visit their house with rations, and they came and collected it.”* (*Female field staff, TB survivor*)

However, we also heard voices stating that once TB was cured, there was not much to worry about the marriage prospects of PwTB, especially in the rural and tribal areas. Practices to prevent the spread of infection can also be construed as stigma or can give rise to perceived stigma by the patient.

We probed the possible stigma related to ration being delivered, which has implications for programmatic scale-up*.*

*“Nothing like that. Even when brother (field staff) used to come to give food, neighbors used to ask why they were coming. We just told the reason.”* (*Male PwTB)*

## Discussion

The qualitative sub-study of the RATIONS trial reaffirmed the felt need for food-based intervention in PwTB and their household contacts. There are valuable additional insights into the possible composition of the food basket, the acceptability, the feasibility, the perceived benefits, and the background conditions like poor nutrition and food insecurity. Many participants alluded to food insecurity, manifesting in the unaffordability of nutrient-dense foods like sattu and milk powder to even cutting back on the number of meals consistent with moderate to severe food insecurity definitions by FAO [25]. Undernutrition is most commonly due to food insecurity, which “is the limited or uncertain availability of nutritionally adequate, safe foods or the inability to acquire foods in socially acceptable ways [26].” The trial had > 80% of the PwTB [10], and > 1/3^rd^ HHCs with undernutrition [9]. The background to this is poverty [16], in which people develop TB, further exacerbated by the inability of the breadwinner to work, which creates a vicious cycle.

Food insecurity is widely prevalent in India [27], including moderate to severe food insecurity in households with PwTB [26,28], which is linked with many adverse outcomes [29]. In the recent comprehensive national nutrition survey, 3 of 5 adolescents did not have access to nutritious foods like fruit or milk even once a week [30]. In the National Family Health Survey-5, more than half of adults did not consume any pulses daily, nearly half did not consume any dairy products or dark green vegetables, more than 80% did not consume any fruits, and more than 90% did not consume any eggs or flesh foods [31]. Household food insecurity was strongly associated with the development of active TB in children (aOR: 11.55) [29]. Food insecurity is also linked to treatment adherence [32], and clinical outcomes in PwTB, with a fivefold higher risk of death [29]. The need for a food basket in such a situation was evident by the term “life-saving” used by some trial participants. Food insecurity is linked to a higher prevalence of mental health issues such as depression and anxiety and impacts treatment success [33].

The acceptability of the food baskets was high and attributed to content and quality. The perceptions about their quantity and duration varied according to individual needs and lived experiences. The composition of the trial food basket was based on the NTEP policy document [13]. Similar food baskets based on dry rations containing cereals and pulses have been well accepted in West Bengal and Madhya Pradesh in India [34–36]. Also, food baskets have been used with good acceptability in Afghanistan [37], Senegal [38], Angola [39], and Brazil [40]. These perspectives and in-depth understanding of “*why things are the way they are rather than how much they are a certain way*” are critical in guiding global recommendations and policymakers [41]. In India, 75% of rural and 50% of rural households have access to cheaper rice and wheat in PDS, which meets the cereal requirements and assumes that the resultant savings will improve their non-cereal intake [42]. Patients appreciated the quality and diversity of the food basket with the versatility of sattu that can be made into a savory or sweet drink, rolled into sweet balls, or stuffed into freshly baked savory Indian bread (Paratha). The food basket breaks the monotony as there is a choice of recipes, which is not the case with ready-to-use therapeutic foods (RUTF), where people may not like the taste or find a preparation too sweet or salty [38]. Locally available and acceptable sources of proteins like sattu need to be identified for scale-up.

The perceived “positive experience” of the food baskets included being able to return to work. Moreover, better adherence, reduced mortality, and reduced side effects are important to patients and NTEP. A very striking feature in Indian PwTB patients is the high prevalence and severity of undernutrition [10–12,43]. Undernutrition and poor weight gain are associated with a higher risk of death, drug toxicity, and recurrence of TB even after a cure [5,44,45]. With severe weight loss and poor weight gain, the ability to return to work is impaired. The persistence of undernutrition (40% continued to remain underweight at 6 months in the trial because of severe undernutrition at baseline) [10], and the presence of post-TB sequelae explains why some patients reported an inability to work as usual at the end of treatment [46]. This observation has an important policy implication regarding extended support for severely underweight patients and the necessary resource planning and program implementation.

Food baskets served as an enabler to better adherence to drugs that were otherwise difficult to tolerate, which has been observed in a systematic review [47]. The role of food insecurity in increasing the frequency and intensity of adverse drug reactions (ADR) has been noted in other countries, including in patients with HIV disease [32,48]. Undernutrition is a risk factor for drug toxicity with anti-TB drugs, but there is a paucity of ADR studies on PwTB with undernutrition as a comorbidity. A cohort of tribal patients in a community with a high burden of TB and undernutrition documented ADR in nearly 90% of PwTB, much more than that in our trial [10,49]. In a recent study in children and adolescents, ADRs were strongly associated with malnutrition, with Grade-3 ADRs exclusively seen in patients with malnutrition [50].

The RATIONS trial used food-based nutritional support and had high levels of acceptability among the beneficiaries. Acceptability in the intended beneficiaries is crucial in an intervention likely to involve investments of manpower, money, and time. There are discussions about introducing peanut-based energy-dense nutrition supplements (EDNS)/RUTF in India for PwTB [51]. A study of the acceptability of peanut-based EDNS in 102 PwTB over a 2-month period reported a 10% refusal and 14% adverse effects, and it provided 60 gm fat and 28 gm protein per day [52]. Unfortunately, this would not address the additional protein requirement in TB and the lost lean body mass. Also, the high-fat content may result in nausea and fat accretion rather than lean mass. In a small study in Africa of RUTF vs. food baskets in PwTB, nearly half reported a preference for food baskets [38]. In studies with peanut-based RUTF in Bangladesh, 40% of caregivers of children and nearly 80% of women found it unacceptable as a food product, citing palatability and smell despite the perception of a therapeutic benefit [53,54]. In Indian children, the acceptability of a ‘khichri’ (cooked preparation of rice and legumes) was much higher than that of a peanut-based RUTF [55]. RUTFs risk mystifying nutrition and making local communities dependent on higher-priced externally sourced food items. In 2015, a nutritional support program for patients with multi-drug-resistant tuberculosis in Mumbai had a 58% refusal rate as patients were not happy with the ready-to-eat foods offered, and the authorities switched to a food-based intervention [56].

The issue of the duration of food baskets and other alternative contents reflected a nuanced understanding of the feasibility of support. There is a need for an extension of support when the baseline weights are very poor, and there is a physiological limit to necessary weight gain over the treatment period [10].

Regarding the feasibility of implementing nutritional support, there was strong support from the community health workers with the caveat of community involvement and supportive supervision of the process. Additionally, the Sahiyas handed over a written petition at the end of FGD to convey this wish to the NTEP. The trial field staff provided valuable insights into the challenges of making doorstep deliveries of food rations in challenging terrain and hard-to-reach areas. A recent modeling study emphasizes the cost-effectiveness of food-based intervention within a feasible budget with improvement in equity and reducing the TB burden among those who are susceptible [57].

The interviews explored cash transfers, which are an operationally easier alternative to facilitate purchasing nutritious foods and have been operational since 2018 as NPY [23]. Some challenges in cash transfers include patients from the unorganized sector, those depending on daily wages, with no paid sick leave, continued wages, or non-availability of comprehensive health insurance covering outpatient and inpatient care [58]. Earlier studies have shown significant levels of indebtedness, mainly due to indirect costs associated with loss of wages in Indian PwTB [59]. In a recent national cost survey, > 45% of PwTB incurred catastrophic costs (costs >20% of annual household income) [60]. The question of cash transfers vs. food support in kind drew responses that either supported in-kind food support or the provision of both food and cash. The amount of cash in a cash transfer-only model should be adequate for purchasing a nutritious diet with adequate accounting for food inflation. An impact evaluation of the NPY has shown that non-receipt of DBT was associated with more than 4-fold higher odds of unfavorable outcomes [61]. As a positive step, the cash benefit for PwTB is to be doubled [51]. A vital step should be assessing the nutritional impact of these initiatives to shape future policies.

Finally, the qualitative study highlighted some persistent misconceptions that can impact rational and cost-effective nutritional care: the desire to buy branded sugary energy powders with little value for money. This can be attributed to the aggressive marketing of such products in print and audio-visual media, reinforcing the need to add counseling as an important long-term enabler in TB care [7,62].

### Strengths and limitations

This qualitative inquiry had numerous strengths. First, it represents voices often under-represented in the literature because they are rural, live in remote areas, and belong to indigenous communities. Second, when qualitative aspects are studied in trials, there is potential bias when studied by the trial investigators. We minimized it by involving social scientists unrelated to the trial. Third, we ensured that the researchers or the field staff who knew the participants were not present during the interviews/discussions. Fourth, in-person interactions at the homes of the trial participants helped shape the understanding of the background conditions, the terrain, and the non-verbal communication that can be affected by online methods. Lastly, interim findings from the quantitative trial component were available to shape the interview guide.

The study had some limitations. The purposive sampling may not have fully represented the spectrum of perceptions, experiences, and opinions. The interviews were conducted primarily in Hindi, and some of the nuances expressed in the local language may have been affected. We did not explore gender issues and the experiences of children and adolescents. Lastly, the sample size of PwTB and HHCs could be considered small compared to the large trial population. However, given the background of poverty and food insecurity exacerbated by TB disease as well as the COVID pandemic in a relatively homogenous population, the interviewers perceived data saturation, a concept studied in the past [63].

### Implications for policy, practice, and research

Undernutrition is a recognized and serious comorbidity in Indian PwTB and the most prevalent risk factor for TB incidence in India. India was one of the first countries to adapt the WHO guidelines for nutritional care and support and launched DBT to enable better nutrition for PwTB. The quantitative component of the RATIONS trial, as well as the qualitative inquiry discussed here, are relevant for the NTEP as the program plans to enhance the cash transfer, introduce appropriate nutritional support in kind, as well as expand food baskets to cover TB-affected households. This study has provided insights, perceptions, and experiences of beneficiaries and stakeholders, and evidence of how this food-based intervention can be therapeutic for patients, preventive for contacts, provider of social protection in a setting of food insecurity, and an enabler for adherence to therapy, and return to work.

## Conclusions

The qualitative inquiry, the first of its kind, related to food-based intervention in TB-affected households, reflected the voices and lived experiences of poverty, insecurity, and TB, as well as of the stakeholders serving them. These are relevant to framing policy and practice in the NTEP and any future research in this domain. The food-based intervention for the PwTB and their HHCs was perceived as acceptable, feasible, and beneficial for the recipients and the NTEP. The lived experiences expressed as weight gain, recovery of strength to work, and ability to adhere to treatment because of better tolerability of drugs are in line with “patient-centered” outcomes aligning with India’s resolve to end TB and the global END TB strategy. Opinion on cash or support in kind was divided; many preferred food support over cash, but others expressed a requirement for both. Beneficiaries had a nuanced understanding of the limitations of the government, but extending coverage to household members and duration of nutrition support when required was suggested.

## Supporting information

S1 Table Demographic characteristics of study participants and participant type in the qualitative sub-study of the RATIONS trial. (DOCX)

S1 Text Guidelines and steps followed for the In-depth Interviews. (DOCX)

S2 Text Guidelines and steps followed for the Focus Group Discussions. (DOCX)

S1 Checklist COREQ Checklist. (PDF)
