# Supplementary material for: Qualitative study of acceptability, benefits, and feasibility of a food-based intervention among participants and stakeholders of the RATIONS trial
Source: PLOS Glob Public Health. 2025 Apr 28;5(4):e0004219. doi: 10.1371/journal.pgph.0004219 (PMC12036838; doi:10.1371/journal.pgph.0004219)
Supplement: S1 Table — (DOCX) [file pgph.0004219.s001.docx]

**Supplementary Table 1: Demographic characteristics of study participants and participant type in the qualitative sub-study of the RATIONS trial**

| **Participant Characteristics** | **Patient with TB (12)** | **Household contacts (6)^#^** | **RATIONS field staff (16)** | **Sahiyas (18)** | **RATIONS Project consultants (2)** | **NTEP Staff (4)** |
| --- | --- | --- | --- | --- | --- | --- |
| Age group |  |  |  |  |  |  |
| 18-29 (13) | 3 | 2 | 3 | 5 | - | - |
| 30-39 (32) | 5 | 1 | 13 | 10 | 1 | 2 |
| 40-49 (4) | 2 | 1 | - | 1 | - | - |
| 50-60 (9) | 2 | 2 | - | 2 | 1 | 2 |
| Sex |  |  |  |  |  |  |
| Male (34) | 9 | 4 | 15 | - | 2 | 4 |
| Female (24) | 3 | 2 | 1 | 18 | - | - |
| Education |  |  |  |  |  |  |
| Not adequately literate (6) | 4 | 2 | - | - | - | - |
| Primary school (5) | 4 | 1 | - | - | - | - |
| High school (21) | 3 | 1 | - | 17 | - | - |
| Graduate (16) | 1 | 1 | 13 | - | - | 1 |
| Post Graduate (8) | - | - | 3 | - | 2 | 3 |
| Not mentioned (2) | - | 1 | - | 1 | - | - |
| Occupational status |  |  |  |  |  |  |
| Unemployed (8) | 6 | 2 |  |  |  |  |
| Employed (50) | 6 | 4 | 16 | 18 | 2 | 4 |
| Type of data collection |  |  |  |  |  |  |
| In-depth interview (19) | 9 | 4 | - | - | 2 | 4 |
| Focused group discussion (4) | - | - | 2 | 2 | - | - |
| Group interview (3)* | 3 | 2 | - | 1 | - | - |

^#^2 Household Contacts were also Sahiyas; *3 group interviews were done with PwTB along with their HHC, one of whom was a Sahiya
