# Supplementary material for: Qualitative study of acceptability, benefits, and feasibility of a food-based intervention among participants and stakeholders of the RATIONS trial
Source: PLOS Glob Public Health. 2025 Apr 28;5(4):e0004219. doi: 10.1371/journal.pgph.0004219 (PMC12036838; doi:10.1371/journal.pgph.0004219)
Supplement: S1 Text — (DOCX) [file pgph.0004219.s002.docx]

**Supplementary Appendix 1:**

**Guidelines and steps followed for the In-depth Interview (IDI)**

**We followed these steps for conducting IDI:**

1. Preparation
   1. Ensuring the time and location of IDI and tools needed (e.g., recorder, stationery, seating arrangements)
   2. Ensuring that the participant meets the inclusion criteria
   3. Ensuring the absence of a trial team member who is known to the participant
2. Highlights of the process of informed consent-seeking
   1. Introducing the research team
   2. Giving appreciation for participation in this study
   3. Identifying the name, age, and background of participants (patients/HHC/any other stakeholder). Relevant information on education, occupation/professional experience as relevant
   4. Explaining the study, informed consent, and purpose of the interview
   5. Allowing time for reading and signing the consent
   6. Information about confidentiality of the recording and use only for a scientific purpose.
   7. Information about the average interview time, 90 to 120 minutes
   8. General explanation about how the interview will be conducted, including an emphasized statement that the participants may end the interview anytime
   9. Allowing time for prospective participants to seek clarifications from and ask questions
3. Exploring RATIONS trial participation experiences:

(any of these can be asked first, modified as per the study participants: PwTB, HHC, project consultant, NTEP staff)

1. Can you tell me about your experience as a patient with TB/household contact of PwTB/RATIONS project consultant/NTEP staff
2. Can you tell me about the food basket that you received during treatment
3. Tell me about your main problems/challenges during your treatment/treatment of your household member with TB
4. [Probes (as per the type of study participants) and asked only when necessary]:

- Did you like it? What did you like about it the most?
- How do you compare it to the usual food you eat?
- How did it benefit?
- What else would you have liked in the basket?
- Was it enough?
- Is it feasible to scale up? What could be the challenges to scaling up?
- COVID-19 and (role of) food baskets
- Tell us about stigma due to TB and stigma related to food basket delivery

1. Closing pointers of communication
   - 1. Seeking and encouraging research participants to offer additional comments they wish to share.
     2. Thanking the participant for their participation in this study
