# Supplementary material for: Qualitative study of acceptability, benefits, and feasibility of a food-based intervention among participants and stakeholders of the RATIONS trial
Source: PLOS Glob Public Health. 2025 Apr 28;5(4):e0004219. doi: 10.1371/journal.pgph.0004219 (PMC12036838; doi:10.1371/journal.pgph.0004219)
Supplement: S2 Text — (DOCX) [file pgph.0004219.s003.docx]

**Supplementary Appendix 2:**

**Guidelines and steps followed for the Focus Group Discussions (FGD)**

**We followed these steps for conducting the FGD:**

1. Preparation
   1. Ensuring the time and location of FGD and tools needed (e.g., recorder, stationery, circle seating arrangements) in Panchayat Bhawan or district headquarters with necessary permissions and approvals.
   2. Ensuring the criteria of the participant and the number of participants for the FGD
   3. Ensuring the absence of any person whose hierarchy can affect the response:
      - lead investigators in the case of field staff FGD
      - government officers in the case of Sahiya FGD.
   4. Ensuring appropriate non-hierarchical seating arrangement
2. Highlights of the process of informed consent-seeking
   1. Introduction of the moderator, assistant moderators, and the notetaker
   2. Appreciation for agreeing to participate
   3. Explaining the background of the study and the objective of the FGD
   4. Explaining the informed consent process and documentation, and the purpose of the group discussion
   5. Allowing time for reading, responding to any queries, and signing the consent
   6. Explaining the guidelines of FGD:
   - That there are no correct answers
   - Information about the confidentiality of the recording and use only for scientific purposes.
   - Information on the frame of the discussion: 120 to 180 minutes and that a participant can leave anytime
   - The participants do not need to agree with other participants
   - Importance of listening to others and their views
   1. Information that there will be an opportunity for the questions
3. General Questions

(any of these can be asked first, modified as per the study participants: field staff, Sahiya)

1. Can you tell me about your role/activities related to TB
2. What do you know about the RATIONS project, or how are you engaged in this project?
3. Can you tell me about the food basket that you delivered/saw being delivered during TB treatment
4. Tell me about your main problems/challenges of RATIONS-like food-based intervention
5. [Probes (as per the type of study participants in the FGD) and asked only when deemed necessary for elaborate answers]:
   - - Did you come across complaints/refusal/reluctance for the food basket
     - Your views regarding if food baskets made a difference to patients who received and how and who did not receive food baskets (especially for FGDs with Sahiyas)
     - Challenges during COVID-19, lock-down
     - Disruption of TB services during COVID-19
     - Positive experiences that you remember
     - According to the food habits of the community, what do you think should have been the contents of the food basket
     - How could this have been done differently?
     - Stigma related to TB: your understanding and experiences
     - Interaction of Sahiyas with field staff and field staff with Sahiyas
     - Relationship with other stakeholders in the NTEP
6. Closing statement
   - - - Offering additional comments or questions related to the interview or study
       - Confirming the important notes/findings to the participant
       - Expressing thanks for participation in this study
